# Structural and Functional Peculiarities of Cytoplasmic Tropomyosin Isoforms, the Products of *TPM1* and *TPM4* Genes

**DOI:** 10.3390/ijms22105141

**Published:** 2021-05-13

**Authors:** Marina Marchenko, Victoria Nefedova, Natalia Artemova, Sergey Kleymenov, Dmitrii Levitsky, Alexander Matyushenko

**Affiliations:** 1 Research Center of Biotechnology, A.N. Bach Institute of Biochemistry, Russian Academy of Sciences, 119071 Moscow, Russia; marchenko_m@mail.bio.msu.ru (M.M.); viktoriya-neff@mail.ru (V.N.); ximikk@gmail.com (N.A.); levitsky@inbi.ras.ru (D.L.); 2 Department of Biochemistry, School of Biology, Moscow State University, 119234 Moscow, Russia; 3 Koltzov Institute of Developmental Biology, Russian Academy of Sciences, 119334 Moscow, Russia; s.yu.kleymenov@gmail.com

**Keywords:** tropomyosin, cytoplasmic isoforms, actin filaments, circular dichroism, differential scanning calorimetry

## Abstract

Tropomyosin (Tpm) is one of the major protein partners of actin. Tpm molecules are *α*-helical coiled-coil protein dimers forming a continuous head-to-tail polymer along the actin filament. Human cells produce a large number of Tpm isoforms that are thought to play a significant role in determining actin cytoskeletal functions. Even though the role of these Tpm isoforms in different non-muscle cells is more or less studied in many laboratories, little is known about their structural and functional properties. In the present work, we have applied various methods to investigate the properties of five cytoplasmic Tpm isoforms (Tpm1.5, Tpm 1.6, Tpm1.7, Tpm1.12, and Tpm 4.2), which are the products of two different genes, *TPM1* and *TPM4*, and also significantly differ by alternatively spliced exons: N-terminal exons 1a2b or 1b, internal exons 6a or 6b, and C-terminal exons 9a, 9c or 9d. Our results demonstrate that structural and functional properties of these Tpm isoforms are quite different depending on sequence variations in alternatively spliced regions of their molecules. The revealed differences can be important in further studies to explain why various Tpm isoforms interact uniquely with actin filaments, thus playing an important role in the organization and dynamics of the cytoskeleton.

## 1. Introduction

Tropomyosin (Tpm) is widely represented in all animal organisms and presented in all their tissues [1]. Tpm molecule is an *α*-helical coiled-coil protein dimer, which forms a continuous head-to-tail polymer along the surface of actin filament [2,3]. Despite the apparent simplicity of its structure, Tpm can perform many functions. Tpm participation was shown for such vital processes as morphogenesis and tissue differentiation [4,5], vesicle transport within the cell [6], cellular adhesion [7,8], regulation of skeletal and cardiac muscle contraction [2,3,9]. Moreover, Tpm regulates the stability of actin filaments [10,11] and their dynamics [12,13,14].

At present, about 40 isoforms of Tpm were found in mammals [1]. It seems likely that a variety of Tpm isoforms is closely related to the plethora of Tpm functions. There are four mammalian Tpm genes (*TPM1*, *TPM2*, *TPM3*, and *TPM4*), which give rise to various Tpm isoforms by alternative splicing of exons 2, 6 and 9 [1,2,3,15,16]. Based on their molecular weight, these isoforms have been divided into two large groups: Tpm isoforms of high molecular weight (HMW) that are encoded by 9 exons and typically contain ~284 amino acid residues, and low molecular weight (LMW) isoforms that do not utilize exon 2 and consist of ~247 residues in their primary structure [16].

**Figure 1:**
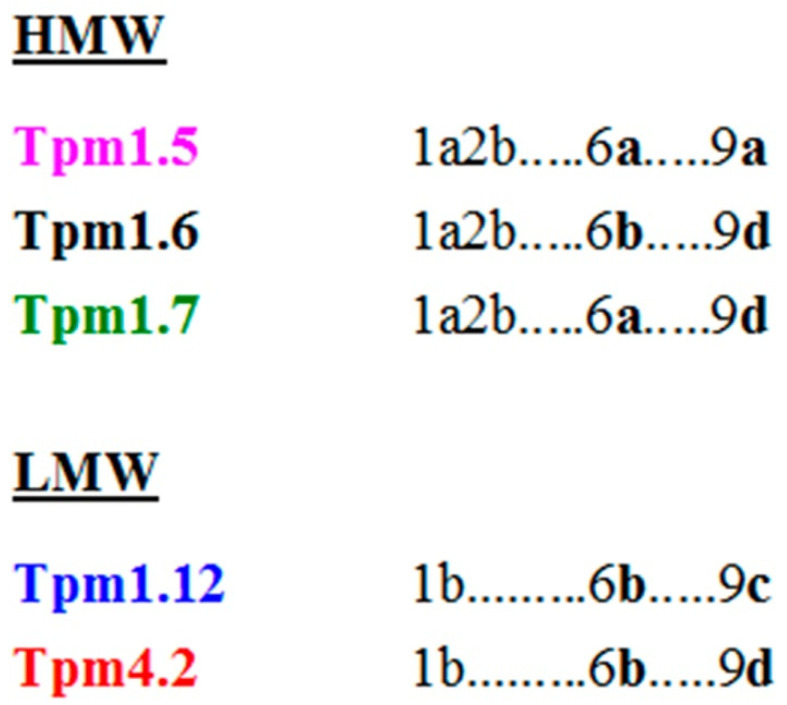
Schematic representation of exon usage for Tpm isoforms used in this study. Variable exons 6 (6a or 6b) and 9 (9a, 9c or 9d) are only shown.

In the present study, we compare structural and functional properties of 3 HMW isoforms, the products of the *TPM1* gene (Tpm1.5, Tpm 1.6, and Tpm1.7), with those of 2 LMW isoforms, one of which (Tpm1.12) is encoded by the *TPM1* gene and another (Tpm4.2) is the product of the *TPM4* gene. The differences between HMW isoforms are caused by variable exons 6 (6a or 6b) and 9 (9a or 9d), while LMW isoforms Tpm1.12 and Tpm4.2 produced from different genes differ from each other not only by alternatively spliced exon 9 (9c or 9d) (Figure 1) but also by constitutive exons [15].

Most of these Tpm isoforms are known to be spatially and developmentally expressed in neuronal cells, including several brain-specific isoforms [17,18,19], some of which, such as a major neuronal isoform Tpm1.12, are always expressed in all brain regions and involved in neuronal morphogenesis, while others (e.g., Tpm1.6 and Tpm1.7) are only present in embryonic brain [20]. At the same time, almost nothing is known yet on physiological functions of Tpm1.5 isoform, except only that it is found to be expressed, among other Tpm isoforms, in mouse lens epithelial cells [21]. As to the *TPM4* gene product Tpm4.2, it is widely distributed in various non-muscle cells [22]. This Tpm isoform was found in the brain where it was concentrated in the growth cones of cultured neurons and, in vivo, in areas where neurites were growing [23]. Tpm4.2 decoration of actin filaments was shown to stimulate the actin-induced ATPase activity of non-muscle myosin IIa [24], thus suggesting an important role for this Tpm isoform in regulating myosin functions.

Although the role of these Tpm isoforms in different compartments of mature and developing neuronal cells is more or less studied in many laboratories [17,18,19,20,22,23,24], much less is known about their structural and functional properties. In particular, it was shown that cooperativity of actin binding was 3-fold higher for HMW Tpm isoforms Tpm1.6 and Tpm1.7 than for LMW isoforms Tpm1.12 and Tpm4.2 [25]. However, highly contradictory results were obtained in different laboratories in studies of the binding of the Tpm1.12 isoform to F-actin [25,26,27]. In some cases, the actin affinity of this Tpm isoform was rather low [26] or even extremely low [27], while in the other study normal affinity was observed similar to those for other Tpm isoforms [25].

Recently we applied differential scanning calorimetry (DSC) to investigate thermal unfolding and domain structure of various non-muscle human Tpm isoforms, including HMW isoforms Tpm1.5, Tpm1.6, and Tpm1.7, which are significantly different by alternatively spliced exons 6 and 9, as well as LMW isoform Tpm1.12 [28]. The results demonstrated that the structural properties of the Tpm isoforms can be quite different depending on the presence of different alternatively spliced exons in their genes.

Our aim in the present research was to extend these studies and to determine how the alternatively spliced exons affect functional properties of various cytoplasmic Tpm isoforms produced from two different genes, *TPM1* and *TPM4*. For this purpose, we applied various methods to analyze the properties of 4 Tpm isoforms produced from the *TPM1* gene (HMW isoforms Tpm1.5, Tpm1.6, and Tpm1.7, as well as LMW isoform Tpm1.12), and LMW isoform Tpm4.2, the product of the *TPM4* gene.

## 2. Results

### 2.1. Thermal Stability of Cytoplasmic Tpm Isoforms (CD and DSC Studies)

**Figure 2:**
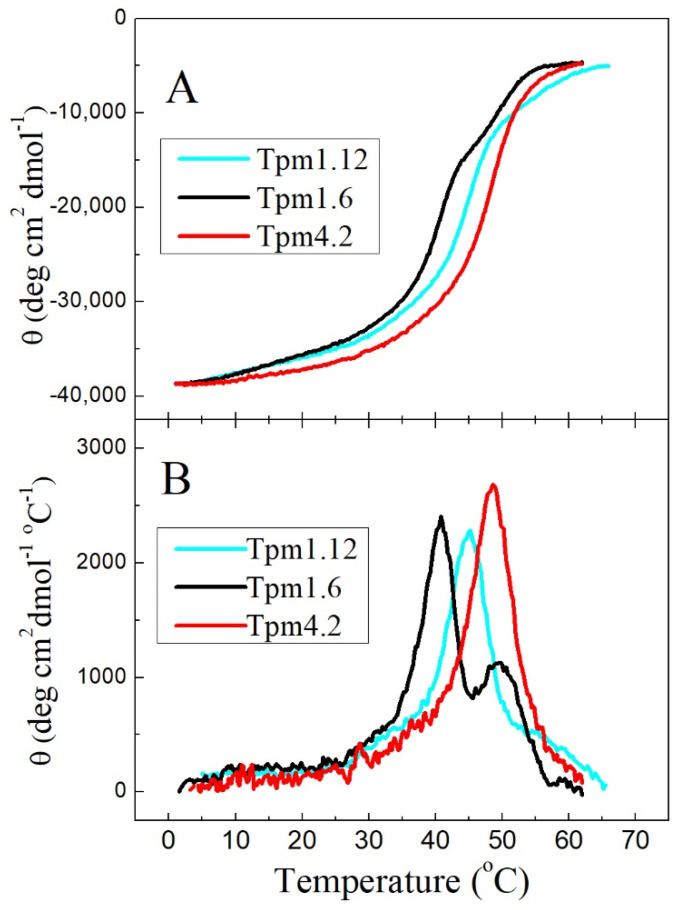
Thermal unfolding of Tpm isoforms Tpm1.6, Tpm1.12, and Tpm4.2 measured by CD. (**A**) Temperature dependences of *α*-helix stability measured at 222 nm. (**B**) First derivative profiles for data shown in (**A**). All experiments were done at the constant heating rate of 1 °C/min with a protein concentration 1 mg/mL.

First, we applied circular dichroism (CD) to study the thermal stability of Tpm isoforms Tpm1.6, Tpm1.12 and Tpm4.2, which were not studied by this method in the previous work [28]. The CD spectra recorded at 5 °C were practically identical for all these Tpm samples and showed two negative peaks at 208 and 222 nm typical of *α*-helical coiled-coil proteins. The thermal unfolding of these Tpm species was examined by measuring the ellipticity at 222 nm, which reflects the *α*-helix content of the Tpm molecule (Figure 2). The results showed that the thermal stability of Tpm4.2 was noticeably higher than that for Tpm1.6 and Tpm1.12 (Figure 2A). The main thermal transition was observed at 48.6 °C for Tpm4.2, at 41 °C for Tpm1.6, and at 45 °C for Tpm1.12 (Figure 2B). Moreover, the Tpm4.2 isoform did not demonstrate a peak at ~50 °C or a shoulder above 50 °C, which were characteristic for Tpm1.6 and Tpm1.12, respectively (Figure 2B) and were assigned in the previous DSC study to the thermal unfolding of the N-terminal part of these Tpm isoforms [28].

**Figure 3:**
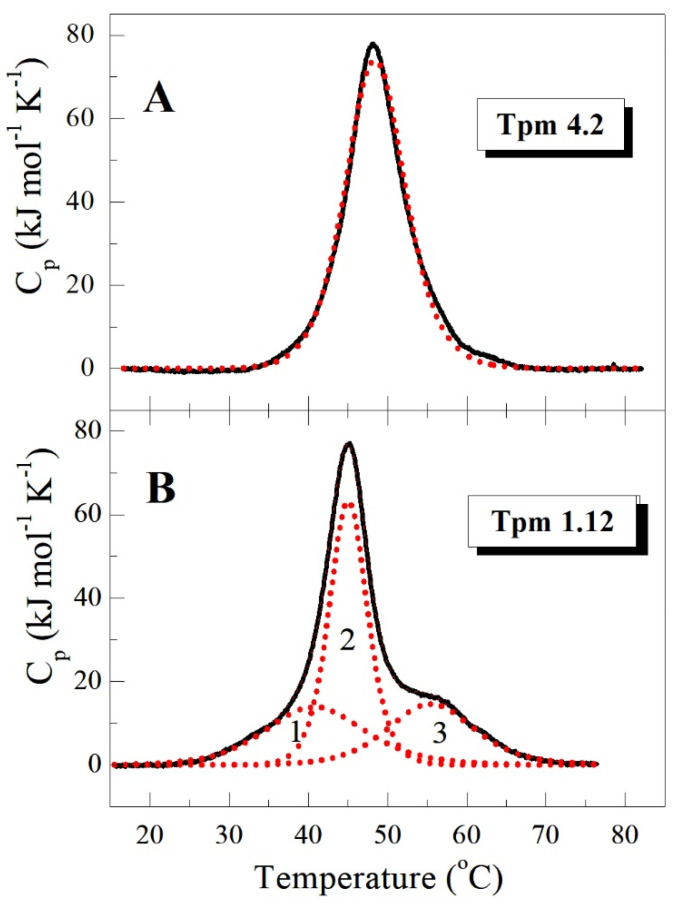
Temperature dependences of the excess heat capacity (Cp) monitored by DSC and the results of the deconvolution analysis of the heat sorption curves for LMW Tpm isoforms Tpm4.2 (**A**) and Tpm1.12 (**B**). Solid black lines represent the experimental curves after subtraction of instrumental and chemical baselines, and dotted red lines represent the individual thermal transitions (calorimetric domains) obtained from fitting the data to the non-two-state model.

To study the thermal unfolding and domain structure of the Tpm4.2 isoform in detail, we applied DSC as a basic method for the investigation of protein thermal unfolding. An important advantage of this method is that it allows one to decompose a heat sorption curve into separate thermal transitions (calorimetric domains) corresponding to the melting of different parts of the Tpm molecule, and thereby to explore specific features of its structure. This DSC approach has been successfully used in our previous study where we analyzed and compared the thermal unfolding and domain structure of different non-muscle Tpm isoforms belonging to both HMW group (Tpm1.5, Tpm1.6, and Tpm1.7) and to LMW group (Tpm1.12) [28]. Although these Tpm isoforms significantly differed in their domain structure, they all demonstrated three calorimetric domains on the DSC curve, and the most thermostable domain being assigned to the thermal unfolding of the N-terminal part of their molecules. In contrast, the Tpm4.2 isoform studied here demonstrated the only calorimetric domain on its DSC curve, with a transition temperature (*T*_m_) of 48.0 °C and calorimetric enthalpy (ΔH_cal_) equal to 750 kJ/mol (Figure 3A).

In this regard, the Tpm4.2 isoform significantly differs from other non-muscle Tpm isoforms earlier studied by DSC, which always demonstrated three calorimetric domains on the DSC profile [28]. As an example, Figure 3B shows the thermal unfolding of LMW Tpm1.12 isoform demonstrating 3 calorimetric domains on its DSC profile.

### 2.2. Interaction between the N- and C-Ends of Tpm Isoforms

Neighbouring Tpm molecules can interact by their ends, forming a continuous head-to-tail polymer, which is located along the surface of actin filament and plays a crucial role in the regulation of actin-myosin interaction. To assess the ability of various Tpm isoforms to form a long strand, we measured the viscosity of their solutions. The measurements were carried out at different Tpm concentrations (1 and 2 mg/mL), and the excess viscosity values obtained after subtraction of buffer viscosity (1.0412 mPa∙s) are presented in Table 1 as mean ± S.D. for three experiments.

The results showed that the viscosity of Tpm isoforms belonging to HMW group (Tpm1.5, Tpm1.6, and Tpm1.7) was much higher compared with the isoforms of the LMW group (Tpm1.12 and Tpm4.2) (Table 1). Among HMW isoforms, the highest viscosity was observed for Tpm1.7, indicating enhanced head-to-tail interactions between adjacent molecules of this Tpm isoform in the overlap region, while Tpm1.5 demonstrated the least viscosity. As to LMW isoforms, the viscosity of Tpm1.12 was substantially less, by 2.5–3 times, than that of Tpm4.2 (Table 1).

### 2.3. Affinity of Tpm Isoforms for F-actin

**Figure 4:**
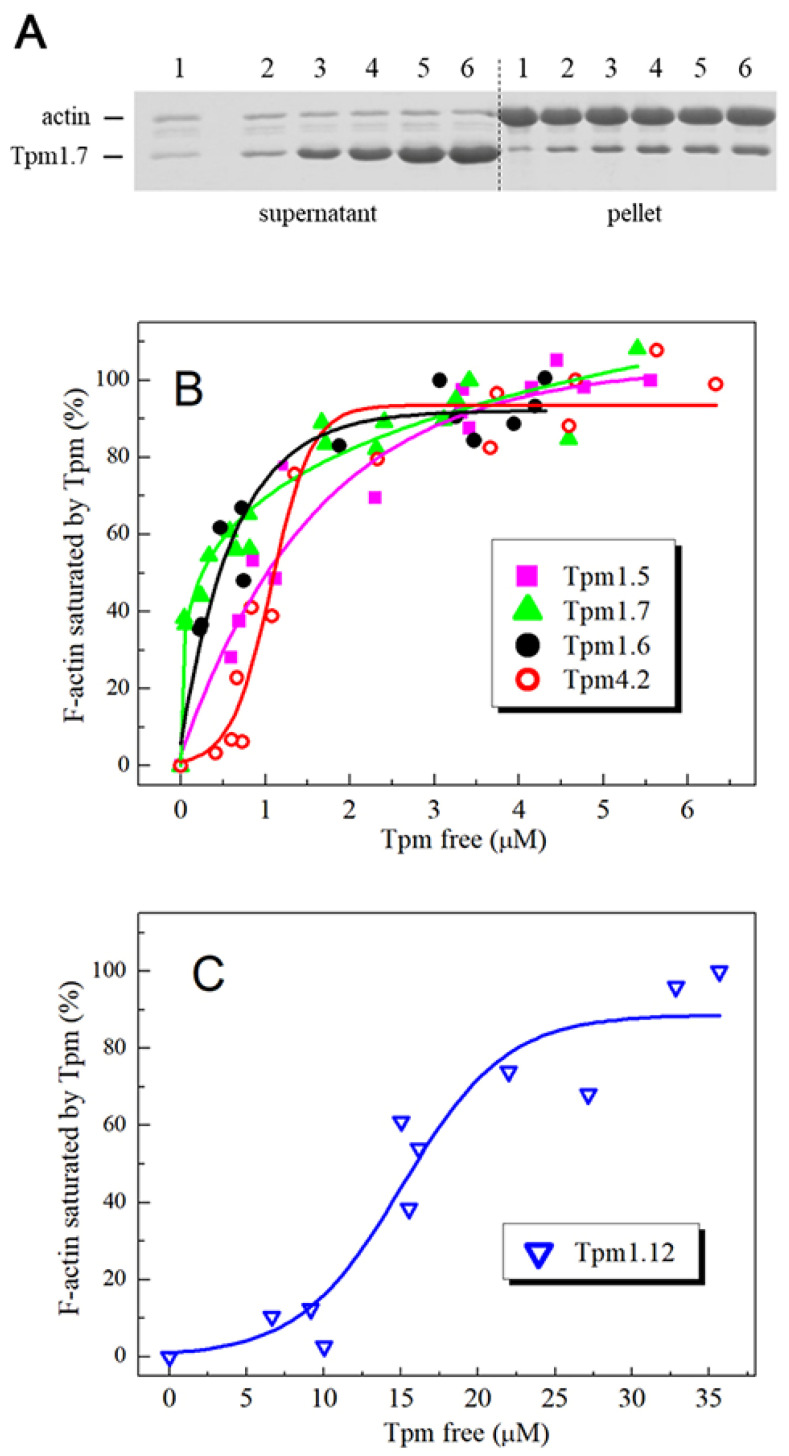
The affinity of various Tpm isoforms for F-actin. Plot (**A**) shows a representative SDS-PAGE gel for experiment of cosedimentation of Tpm1.7 isoform with F-actin. The numbers 1–6 above the lanes for supernatants and pellets correspond to concentrations of Tpm1.7 (0.5 μM, 1 μM, 2 μM, 3 μM, 4 μM, and 6 μM, respectively) added to F-actin before ultracentrifugation. Plots (**B**) and (**C**) show the actin affinity of Tpm isoforms Tpm1.5, Tpm1.6, Tpm1.7, and Tpm4.2 (**B**), and Tpm1.12 (**C**) determined by the cosedimentation assay and plotted as the fractional saturation of F-actin by Tpm as a function of free Tpm concentration that was found in the supernatant.

To investigate the affinity of various cytoplasmic Tpm isoforms for F-actin, we applied the cosedimentation assay (Figure 4). For HMW Tpm isoforms (Figure 4A), the K_50%_ values, corresponding to the Tpm concentration at which actin is half-saturated, varied from 0.4 µM to 1.1 µM for all these Tpm isoforms (Table 2). Among them, the lowest actin affinity was observed with Tpm1.5 for which the K_50%_ value was similar to that obtained for Tpm4.2 isoform from the LMW group (Table 2). However, this LMW isoform bound to F-actin with higher cooperativity than HMW isoforms did (Figure 4A).

The most intriguing result was obtained with the Tpm1.12 isoform, which demonstrated very low affinity for F-actin (Figure 4B), and its K_50%_ value was much higher (by ~15 times) than that for all other Tpm isoforms studied (Table 2).

### 2.4. Thermally Induced Dissociation of the F-actin Complexes with Tpm Isoforms

**Figure 5:**
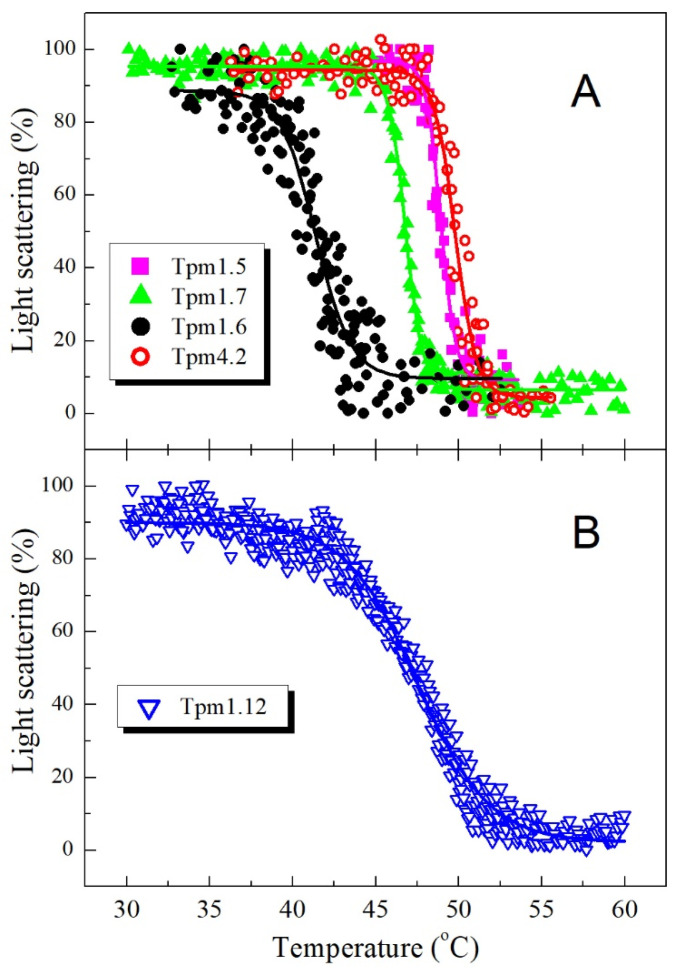
Normalized temperature dependences of dissociation of the complexes of F-actin with Tpm isoforms Tpm1.5, Tpm1.6, Tpm1.7, and Tpm4.2 (**A**), and Tpm1.12 (**B**). A 100% value corresponds to the difference between the light scattering of Tpm–F-actin complexes measured at 25 °C and that of pure F-actin stabilized by phalloidin, which was temperature-independent within the temperature range used. A decrease in the light-scattering intensity reflects the dissociation of the Tpm–F-actin complex. Samples contained 20 μM F-actin stabilized by 20 μM phalloidin and 10 μM Tpm (**A**) or 10 μM phalloidin-stabilized F-actin and 120 μM Tpm1.12 (**B**) in 30 mM Hepes-Na buffer, pH 7.3, containing 100 mM NaCl and 2 mM DTT.

To investigate the stability of the complexes formed by various Tpm isoforms with F-actin, we measured the temperature dependences of their light scattering (Figure 5), whose decrease reflects the thermally induced dissociation of the complexes.

The main parameter extracted from an analysis of dissociation curves is *T*_diss_, that is, the temperature at which a 50% decrease in the light scattering occurs. The *T*_diss_ values obtained for dissociation of the F-actin complexes with various Tpm isoforms can be arranged in the following order: Tpm1.6 < Tpm1.7 < Tpm1.12 < Tpm1.5 < Tpm4.2 (Table 2). Importantly, absolutely the same order can be obtained from DSC data for these Tpm isoforms by a comparison of the temperature maxima of their thermal transitions on the DSC profiles ([28], Figure 3A). Moreover, the Tpm1.12 isoform, whose affinity for actin was extremely low (Figure 4B, Table 2), dissociated from F-actin at a rather high temperature, with *T*_diss_ value of 47.5 °C (Table 2) similar to transition temperature (*T*_m_) on the DSC profile of this Tpm isoform (Figure 3B). It should be noted, however, that due to the very low affinity of Tpm1.12 for F-actin (Figure 4B, Table 2), these experiments could not be performed under standard conditions used for all other Tpm isoforms (at Tpm and F-actin concentrations equal to 10 μM and 20 μM, respectively), and therefore a molar ratio of Tpm to F-actin was significantly increased in this case (120 μM Tpm vs. 10 μM F-actin) (Figure 5B). It should also be noted that this Tpm isoform dissociated from F-actin within a broad temperature range, 15 °C (from 40 to 55 °C) (Figure 5B), which was much wider than the range for all other Tpm isoforms dissociating within a much narrower range of 4–7 °C (Figure 5A). This low cooperativity of Tpm1.12 dissociation from F-actin seems to be caused by changed experimental conditions because of the extremely low affinity of this Tpm isoform for F-actin.

## 3. Discussion

The main aim of our work was to investigate and compare the properties of various cytoplasmic Tpm isoforms, which are the products of two different genes, *TPM1* and *TPM4*, belong to either HMW (Tpm1.5, Tpm1.6, and Tpm1.7) or LMW group (Tpm1.12 and Tpm4.2) and differ from each other by alternatively spliced exons 6 and 9 (Figure 1). We showed that studied Tpm isoforms differ in their stability, the affinity of Tpm for actin filaments, the stability of Tpm complexes with F-actin, and the strength of the head-to-tail interactions of Tpm dimers. These results may be important for physiological studies. We can hypothesize that structural properties of Tpm isoforms may determine which protein partners interact with Tpm strand; the variations in the Tpm affinity for F-actin may discriminate the decoration of F-actin in different cell compartments, as well as the stability of the Tpm-F-actin complex, may be important for the formation of stable or dynamic actin structures inside the cell. All these assumptions require confirmations in future studies. In this work, we considered separately the biophysical and biochemical properties of Tpm isoforms belonging to HMW or LMW groups, which significantly differ by the N-terminal exons, 1a2b in HMW isoforms and 1b in LMW isoforms (Figure 1).

### 3.1. Properties of HMW Isoforms of Non-Muscle Tpm (Tpm1.5, Tpm1.6, and Tpm1.7)

HMW Tpm isoforms differ by variable exons 6 (6a or 6b) and 9 (9a or 9d) (Figure 1). Previous DSC studies have shown that the thermal stability of the C-terminal part of Tpm1.6 with exon 6b is significantly less than that of Tpm1.5 and Tpm1.7 containing exon 6a [28]. This significant decrease in the thermal stability of the C-terminal part of Tpm molecule can be explained by the presence of residues destabilizing the coiled-coil structure in exon 6b in comparison with exon 6a (e.g., Cys190, Asp196, Val200, and Thr201 in exon 6b instead of Val190, Gln196, Met200, and Asp201 in exon 6a) [15]. Thus, it can be concluded that just the exon 6b is mainly responsible for the destabilization of the C-terminal part of Tpm molecule. This conclusion is corroborated by the results of previous DSC studies on LMW isoforms Tpm1.8 and Tpm1.9 which differ internally only by exon 6 (exon 6a in Tpm1.9 and exon 6b in Tpm1.8). It had been shown in these studies that the thermal stability of the C-terminal part of Tpm1.8 is much less than that of Tpm1.9 [29].

Previous studies indicated that the stability of the Tpm–F-actin complexes measured as temperature-induced dissociation of Tpm from F-actin is mainly dependent on the thermal stability of the Tpm molecule rather than on the Tpm affinity for actin [30,31,32]. In particular, it was suggested that thermal denaturation of the coiled-coil structure of the Tpm molecule precedes its dissociation from the surface of actin filament [33]. Therefore, it seems not surprising that the Tpm1.6 isoform, whose molecule is the least thermostable due to the presence of exon 6b, dissociates from F-actin at a much lower temperature than other Tpm isoforms (Figure 5A, Table 2).

The viscosity of the Tpm1.5 solution was several times lower than that of Tpm1.6 and especially Tpm1.7 (Table 1), thus indicating significantly reduced head-to-tail interactions between adjacent molecules of this Tpm isoform in the overlap region. The Tpm1.5 isoform differs from other Tpms studied here by C-terminal exon 9, which is 9a in Tpm1.5 and 9d in Tpm1.6 and Tpm1.7 (Figure 1). This indicates that the exon 9a in Tpm1.5 is mainly responsible for the reduced ability of this Tpm isoform to form a long strand on the surface of an actin filament. As a result, the Tpm affinity for actin estimated by the cosedimentation experiments is decreased for Tpm1.5 compared to Tpm1.6 and Tpm1.7 (Figure 4, Table 2). Relatively high Tpm affinity for actin requires the formation of the overlap junctions, and therefore reduced ability of Tpm1.5 with exon 9a to head-to-tail interactions between adjacent molecules in the overlap region also reduces the affinity of this Tpm isoform for actin.

It is also important to note the structure of the overlap region depending on the exons present in the Tpm molecule has been studied previously [34,35]. Greenfield et al., [34] determined the structure of the overlap region between LMW Tpm N-terminal model peptide (exon 1b) and a smooth/non-muscle Tpm C-terminal peptide (exon 9d). They compared this structure with that of skeletal Tpm1.1 containing N-terminal exon 1a and C-terminal exon 9a and showed that the overlap region in the 1b/9d model is longer than in the 1a/9a model but had a similar orientation of N- and C-termini in both cases [34]. Frye et al., (2010) also analyzed the structure of the overlap region between N- and C-termini of smooth muscle Tpm (Tpm1.3) encoded by exons 1a and 9d [35]. In our work, we studied HMW isoforms that had 1a N-terminal exon and 9a/9d C-terminal exon. However, these isoforms differ from each other not only by C-terminal exon but also by internal exon 6 (6a or 6b). The viscosity measurements indicate that the viscosity of Tpm1.6 is much lower than that of Tpm1.7 (Table 1). These Tpm isoforms with the same N-terminal exons (1a2b) and C-terminal exon 9d differ only by internal exon 6. Based on structural models, the solutions of these isoforms are expected to have similar viscosities. The difference in the viscosities suggests that not only N- and C-terminal exons but also internal exon 6 is very important for head-to-tail interactions between Tpm molecules in the overlap region and may potentially affect the orientation of Tpm ends. These data have a good agreement with observations obtained earlier where mutations S229E inside the exon 7 of the Tpm molecule modulates the function of Tpm overlapping ends [36].

### 3.2. Properties of LMW Isoforms Tpm1.12 and Tpm4.2

LMW Tpm isoforms Tpm1.12 and Tpm4.2 are the products of two different genes, *TPM1* and *TPM4,* and they differ by variable exon 9 (9c in Tpm1.12 and 9d in Tpm4.2) (Figure 1) as well as by constitutive exons [15].

The DSC studies have shown that the thermal stability of Tpm4.2 is noticeably higher than that for other non-muscle Tpm isoforms whose thermal unfolding was investigated by this method in the previous work [28]. Moreover, the Tpm4.2 isoform showing only one calorimetric domain on its DSC curve (Figure 3A) is quite different from all other non-muscle Tpm isoforms which always demonstrated three separate calorimetric domains on the DSC profile, including the isoforms with exon 9d, such as Tpm1.6, Tpm1.7, and Tpm3.1 [28]. There are two possible explanations for this unusual thermal unfolding of the Tpm4.2 molecule. One of them is that all parts of the molecule denature together, forming a single calorimetric domain, and another is that their thermal transitions simply coincide in position on the temperature scale and therefore they cannot be separated by deconvolution analysis of the DSC profile. In any case, this unusual thermal unfolding of the Tpm4.2 molecule is a unique property of this Tpm isoform, the product of the *TPM4* gene.

The viscosity of Tpm solutions was much less for LMW isoforms Tpm1.12 and Tpm4.2 than for HMW isoforms Tpm1.5, Tpm1.6, and Tpm1.7 (Table 1), thus indicating significantly weakened head-to-tail interactions between adjacent molecules of LMW Tpms in the overlap region. This weakening of the head-to-tail interactions of LMW Tpm isoforms can be explained by the difference between LMW and HMW Tpm variants in their N-terminal exons, 1b for LMW Tpms and 1a2b for HMW Tpms (Figure 1). It seems quite possible that just the relatively weak head-to-tail interactions of the LMW isoforms cause their presence in dynamic and mobile cell structures (e.g., in neurites during their development), where rapid rearrangement of the actin cytoskeleton is particularly important. In mobile cell structures, the concentration of Tpm1.12 can be high enough to provide its binding to actin filaments, or some actin-binding proteins may significantly increase the Tpm1.12 affinity for actin to provide full saturation of actin filaments with this Tpm isoform.

It should also be noted that the viscosity of the Tpm1.12 solution was substantially less (by 2.5–3 times) than that of Tpm4.2 (Table 1), thus indicating significantly weakened head-to-tail interactions between Tpm1.12 molecules. This can be explained by the difference between these Tpm isoforms in their C-terminal exon 9, namely 9c for Tpm1.12 and 9d for Tpm4.2 (Figure 1). The exon 9c in human Tpm1.12 is three residues shorter than the exon 9d of Tpm4.2, and six of the ten C-terminal amino acid residues encoded by exon 9c are different from the exon 9d sequence [15]. This difference in the C-terminal residues can impair their interaction with the N-terminal residues encoded by exon 1b which are also different in Tpm1.12 and Tpm4.2 isoforms, thus weakening head-to-tail interactions between Tpm1.12 molecules in the overlap region and making worsen their ability to form a long strand.

It should be noted that the C-terminal nine residues define the actin affinity of unacetylated Tpm [27,37]. This can explain why the Tpm1.12 isoform whose C-terminal residues differ from those of the Tpm4.2 demonstrated not only lower viscosity of its solution (Table 1) but also a dramatically lower affinity for F-actin (Figure 4B, Table 2). Similar very low [26], or even extremely low [27], actin affinity of the Tpm1.12 isoform was observed in earlier studies. However, in the other study, normal actin affinity was observed for Tpm1.12 similar to those for Tpm4.2 and other Tpm isoforms (e.g., Tpm 1.6 and Tpm1.7) [25]. Importantly, it was shown in the last study [25] that cytoskeletal Tpm isoforms bind with similar affinity to cytoskeletal F-actin and skeletal F-actin (i.e., the actin which was used in our work).

We tried to resolve the contradiction in the literature concerning the actin affinity of the Tpm1.12 isoform. The authors of the last published work (Miro Janco et al., [25]) wrote that this contradiction “may reflect subtle differences in the way the experiments were performed in the different laboratories”. Indeed, there were some subtle differences in the conditions of cosedimentation experiments. In particular, we used F-actin stabilized by phalloidin and ionic strength of 200 mM NaCl, while in other works bare F-actin was used in the presence of 150 mM NaCl [25,27]. However, extremely low affinity for actin of Tpm with C-terminal exon 9c was observed not only in our study (Figure 4B, Table 2), but also in the work of Joanna Moraczewska et al., [27] where the authors used slightly other conditions for these experiments. Moreover, in both these works, the affinity of Tpm1.12 for actin was much less than that observed for other Tpm isoforms. This means that the extremely low actin affinity of the Tpm1.12 isoform may depend on some peculiarities of its preparation rather than on the conditions of cosedimentation experiments.

We also considered two other possible explanations for the very low actin affinity of the Tpm1.12 isoform. One of them was that two chains of Tpm1.12 could be cross-linked by the formation of a disulfide bond between Cys-154 residues. Previous studies showed that the interchain cross-linking of Tpm1.1 greatly decreased its affinity for F-actin [38]. However, we have shown, using SDS-PAGE [39] under non-reducing conditions, in the absence of *β*-mercaptoethanol or DTT [33], that Tpm1.12 and all other Tpms containing Cys residues (Tpm1.6 and Tpm4.2) were in the fully reduced state. Another possible explanation for very low actin affinity of the Tpm1.12 was that its C-terminal residues for some reason could not normally interact with non-acetylated N-terminus. To check this assumption, we produced recombinant Tpm1.12 that had an Ala-Ser N-terminal extension mimicking N-terminal acetylation of Tpm [40]. It was shown earlier that recombinant *α*-Tpm (Tpm1.1) expressed in *E. coli* loses N-terminal acetylation and cannot bind actin, while Ala-Ser N-terminal extension fully restores the Tpm ability to interact with actin [40]. We confirmed, using MALDI-TOF mass-spectrometry, the presence of Ala-Ser N-terminal extension in this Tpm.12 sample and performed cosedimentation experiments to investigate its affinity for actin. It turned out that the Ala-Ser N-terminal extension did not affect the low actin affinity of Tpm1.12 and the result was absolutely the same as that presented in Figure 4B. This indicated that the extremely low actin affinity of the Tpm1.12 isoform is exclusively determined by the specific structure of its C-terminal exon 9c but not by some other reasons. In this regard, it is rather difficult to explain the results showed that Tpm1.12 can bind to F-actin with a high affinity similar to that for other Tpm isoforms [25].

Because of the very low actin affinity of the Tpm1.12 isoform, it could fully saturate actin filament only at high Tpm:actin molar ratio (Figure 4B) and therefore it dissociated from F-actin within a broad temperature range (Figure 5B). Nevertheless, the temperature of its half-maximal dissociation was similar to the transition temperature of its thermal unfolding on the DSC profile (Figure 3B). This corroborates our previous observations that the stability of the Tpm–F-actin complexes depends rather on the thermal stability of the Tpm molecule than on the Tpm affinity for actin [30,31,32].

## 4. Materials and Methods

### 4.1. Protein Preparations

All Tpm species used in this work were recombinant proteins whose coding sequences were commercially synthesized in Evrogen company (Moscow, Russia) and cloned into pet23a^+^ vector. The HMW Tpm isoforms (Tpm1.5, Tpm1.6, and Tpm1.7) had an Ala-Ser N-terminal extension to mimic natural N-terminal acetylation of native Tpm [40]. The construct of the LMW Tpm1.12 isoform with the Ala-Ser N-terminal extension was also produced by the same method, especially to check the reason for its very low actin affinity (see Section 3.2 in Discussion). The accuracy of the coding sequences of various Tpm isoforms was verified by DNA sequencing.

All Tpm constructs were expressed in *E. coli* C41(DE3) cells by standard protocol, and then extracted and purified by anion exchange chromatography using a HiTrap QHP column (GE Healthcare, Marlborough, MA, USA) as previously described [30,41]. Tpm concentration was determined spectrophotometrically at 280 nm using an E^1%^ of 2.7 cm^−1^ for Tpm1.5, 1.8 cm^−1^ for Tpm1.6 and Tpm1.7, 2.1 cm^−1^ for Tpm1.12, and 1.6 cm^−1^ for Tpm4.2. According to SDS-PAGE [39], the purity of all these Tpm preparations was no less than 95%. The purified proteins were stored at –80 °C.

All Tpm species containing Cys residues (Tpm1.6, Tpm1.12, and Tpm4.2) were reduced before experiments by heating at 60 °C for 20 min in the presence of 3mM DTT. After such a procedure, all these Tpm samples were in the entirely reduced state as it was shown using SDS-PAGE [39] under non-reducing conditions, in the absence of *β*-mercaptoethanol or DTT [33]. In all experiments, the solution contained 2 mM DTT to prevent disulfide cross-linking between Cys residues in two chains of the Tpm molecule.

Rabbit skeletal muscle actin was prepared by a standard method [42]. F-actin polymerized by the addition of 4 mM MgCl_2_ and 100 mM KCl was further stabilized by adding phalloidin (Sigma Chemical Co., St Louis, MO, USA) in a molar ratio of 1:1.

### 4.2. CD Measurements

Far-UV CD spectra of various Tpm species (1.0 mg/mL) were recorded at 5 °C on a Chirascan CD spectrometer (Applied Photophysics, Surrey, UK) in 0.02 cm cells. Measurements of thermal unfolding were performed as described earlier [28,30], by following the molar ellipticity of Tpm at 222 nm over a temperature range from 5 °C to 70 °C at a constant heating rate of 1 °C/min. All measurements were performed in 30 mM Hepes-Na buffer, pH 7.3, containing 100 mM NaCl and 2 mM DTT. The reversibility of the unfolding–refolding process was assessed by reheating the Tpm sample directly after it had been cooled from the previous temperature scan. The thermal unfolding of all Tpm species was fully reversible.

### 4.3. Differential Scanning Calorimetry (DSC)

DSC experiments were performed as described previously [28] on a MicroCal VP-Capillary differential scanning calorimeter (Malvern Instruments, Northampton, MA, USA) at a heating rate of 1 K/min in 30 mM Hepes-Na buffer, pH 7.3, containing 100 mM NaCl and 2 mM DTT. The protein concentration was 2 mg/mL. The DSC scans obtained from the first heating of the Tpm4.2 and Tpm1.12 samples were omitted and the reversibility of the heat sorption curves was assessed by reheating the sample immediately after it had cooled from the previous scan. The thermal unfolding of these Tpm species was fully reversible and can be considered to be at thermodynamic equilibrium, thus making possible further deconvolution analysis of their DSC curves. The calorimetric traces were corrected for the instrumental background by subtracting a scan with the buffer in both cells. The temperature dependence of the excess heat capacity was further analyzed and plotted using Origin 7.0 software (MicroCal Inc., Northampton, MA, USA). The thermal stability of the proteins was described by the temperature of the maximum of the thermal transition (T_m_), and the calorimetric enthalpy (ΔH_cal_) was calculated as the area under the excess heat capacity function. Deconvolution analysis of the heat sorption curves, that is, their decomposition into separate thermal transitions (calorimetric domains) by fitting the data to the non-two-state model [43], was performed as described earlier [28,31,33,41,44].

### 4.4. Viscosity Measurements

The viscosity measurements were performed on a falling ball micro viscometer Anton Paar AMVn (Anton Paar USA Inc., Ashland, VA, USA) in 0.5 mL capillary at 20 °C. The specific density of the Tpm solutions was measured with an Anton Paar DMA 4500 device (Anton Paar USA Inc., Ashland, VA, USA) and taken into account for accurate viscosity calculation. All measurements were performed at a Tpm concentration of 1.0 or 2.0 mg/mL in a 30 mM Hepes-Na buffer (pH 7.3) containing 100 mM NaCl and 2 mM DTT. The measurements for each Tpm sample were repeated three times, and the obtained values were averaged.

### 4.5. Cosedimentation of Tpm Species with F-actin

The affinity of various Tpm isoforms to actin was estimated by cosedimentation assay as previously described [30,31,33,41]. Briefly, 10 μM phalloidin stabilized F-actin was mixed with increasing concentrations of Tpm, from 0 to 5.5–6 μM for all Tpms except only the Tpm1.12 isoform for which the concentration increased up to almost 40 μM due to its very low affinity to F-actin. The probes with a final volume of 100 μL in 30 mM Hepes-Na buffer (pH 7.3) containing 200 mM NaCl were incubated 40 min at 25 °C, then F-actin was pelleted with any bound Tpm by ultracentrifugation at 100,000× *g* for 40 min (Beckman Airfuge; Beckman Coulter, Fullerton, CA, USA). Equivalent samples of the pellet and the supernatant were subjected to SDS-PAGE [39]. Protein bands were scanned and analyzed with ImageJ 1.45 s software (Scion, Frederick, MD, USA).

### 4.6. Temperature Dependences of Light Scattering

Thermally-induced dissociation of Tpm complexes with F-actin stabilized by phalloidin were detected by changes in light scattering at 90 degrees. This approach aimed for investigation of the stability (thermal stability) of Tpm–F-actin complexes was first described many years ago by Albrecht Wegner [45] and then it was often used in our studies [30,31,33,41]. The experiments were performed at 350 nm on a Cary Eclipse fluorescence spectrophotometer (Varian Australia Pty Ltd., Mulgrave, VIC, Australia) equipped with a temperature controller and thermoprobes. All measurements were performed at a constant heating rate of 1 °C/min. The molar ratio of Tpm to F-actin was 1:2 for all Tpms except only the Tpm1.12 isoform with very low affinity to F-actin, for which the Tpm:actin molar ratio was significantly increased, up to 12:1, to reach full saturation of actin filament. When Tpm dissociated from F-actin during heating, the value of the light-scattering intensity became equal to that of F-actin, because the light scattering of free Tpm molecules was negligible [45]. Thus, a temperature-dependent decrease in light-scattering intensity of the Tpm–F-actin complexes reflects dissociation of Tpm from F-actin. The dissociation curves, with the temperature dependence of light scattering for F-actin alone deducted, were analyzed by using the Origin software (MicroCal, Studio City, CA, USA), according to a sigmoidal decay function (Boltzmann). The main parameter extracted from this analysis is T_diss_, that is, the temperature at which there is a 50% decrease in light scattering occurs.

## 5. Conclusions

The main aim of the present research was to determine how the alternatively spliced exons affect the functional properties of various cytoplasmic Tpm isoforms produced from two different genes, *TPM1* and *TPM4*. The amino acid sequences of these isoforms exhibited variability primarily in the N-terminal region (exons 1 and 2), middle region (exon 6) and the C-terminal region (exon 9). Our results clearly demonstrated that the properties of these Tpm isoforms can be quite different depending on the presence of different alternatively spliced exons in their genes, namely, N-terminal exons 1a2b or 1b, internal exons 6a or 6b, and C-terminal exons 9a, 9c or 9d. The revealed differences between various cytoplasmic Tpm isoforms can be important in further studies to explain why these isoforms, which are spatially and developmentally expressed in non-muscle cells, interact uniquely with actin filaments thus playing a key role in the organization and dynamics of the cytoskeleton.

## Figures and Tables

**Table 1:** Excess viscosities of Tpm isoforms solutions over buffer viscosity.

Tpm Isoform	Viscosity, mP∙s	
	1 mg/mL	2 mg/mL
**HMW**		
Tpm 1.5	0.179 ± 0.005	0.653 ± 0.002
Tpm 1.6	0.474 ± 0.005	1.339 ± 0.001
Tpm 1.7	1.027 ± 0.009	3.575 ± 0.001
**LMW**		
Tpm 1.12	0.026 ± 0.004	0.066 ± 0.001
Tpm 4.2	0.064 ± 0.004	0.193 ± 0.005

The viscosity values are presented as averages ± SD for three experiments. The difference between the values for various Tpm isoforms compared in pairs was statistically significant in all the cases (*p* < 0.001, Student’s *t*-test).

**Table 2:** Actin binding properties of various Tpm isoforms and the thermal stability of Tpm–F-actin complexes.

Tpm Isoform	*K*_50%_ (μM)	*T*_diss_ (°C)
**HMW**		
Tpm 1.5	1.11 ± 0.02	48.92 ± 0.04
Tpm 1.6	0.45 ± 0.08	41.38 ± 0.11
Tpm 1.7	0.40 ± 0.10	46.78 ± 0.02
**LMW**		
Tpm 1.12	14.9 ± 2.0	47.49 ± 0.06 *
Tpm 4.2	1.07 ± 0.07	49.79 ± 0.06

Abbreviations: *K*_50%_ (μM), the Tpm concentration at which actin is half-saturated upon co-sedimentation assay; *T*_diss_ (°C), the temperature of the half-maximal dissociation of Tpm–F-actin complexes, that is, the temperature at which a 50% decrease in the light scattering occurs. The *K*_50%_ and *T*_diss_ values presented are averages ± SD for three experiments. The differences between the K50% values for various Tpm isoforms compared in pairs was statistically significant (*p* < 0.001, Student’s *t*-test) in all cases except only the pairs Tpm1.6 vs. Tpm1.7 and Tpm1.5 vs. Tpm1.12, for which the difference was statistically insignificant (*p* > 0.40). The differences between the Tdiss values for various Tpm isoforms compared in pairs was statistically significant in all the cases (*p* < 0.001, Student’s *t*-test).* Due to very low affinity of Tpm1.12 for F-actin, this experiment was performed under changed conditions, at significantly increased molar ratio of Tpm to F-actin (see the text for details).

## Data Availability

Not applicable.
